# Bacteriophage-encoded lytic enzymes control growth of contaminating *Lactobacillus* found in fuel ethanol fermentations

**DOI:** 10.1186/1754-6834-6-20

**Published:** 2013-02-07

**Authors:** Dwayne R Roach, Piyum A Khatibi, Kenneth M Bischoff, Stephen R Hughes, David M Donovan

**Affiliations:** 1 US Department of Agriculture, Animal Biosciences and Biotechnology Laboratory, Animal & Natural Resources Institute, Agricultural Research Service, Beltsville, MD 20705, USA; 2 US Department of Agriculture, National Center for Agricultural Utilization Research, Agricultural Research Service, Peoria, IL, 61604, USA

**Keywords:** Bacteriophage, Lysin, endolysin, Peptidoglycan, Ethanol, Fermentation, Contamination, Lactic acid bacteria, *Lactobacillus*, Lactobacilli

## Abstract

**Background:**

Reduced yields of ethanol due to bacterial contamination in fermentation cultures weaken the economics of biofuel production. Lactic acid bacteria are considered the most problematic, and surveys of commercial fuel ethanol facilities have found that species of *Lactobacillus* are predominant*.* Bacteriophage lytic enzymes are peptidoglycan hydrolases that can degrade the Gram positive cell wall when exposed externally and provide a novel source of antimicrobials that are highly refractory to resistance development.

**Results:**

The streptococcal phage LambdaSa2 (λSa2) endolysin demonstrated strong lytic activity towards 17 of 22 strains of lactobacilli, staphylococci or streptococci and maintained an optimal specific activity at pH 5.5 and in the presence of ≤ 5% ethanol (fermentation conditions) toward *L. fermentum*. *Lactobacillus* bacteriophage endolysins LysA, LysA2 and LysgaY showed exolytic activity towards 60% of the lactobacilli tested including four *L. fermentum* isolates from fuel ethanol fermentations. In turbidity reduction assays LysA was able to reduce optical density >75% for 50% of the sensitive strains and >50% for the remaining strains. LysA2 and LysgaY were only able to decrease cellular turbidity by <50%. Optimal specific activities were achieved for LysA, LysA2, and LysgaY at pH 5.5. The presence of ethanol (≤5%) did not reduce the lytic activity. Lysins were able to reduce both *L. fermentum* (BR0315-1) (λSa2 endolysin) and *L. reuteri* (B-14171) (LysA) contaminants in mock fermentations of corn fiber hydrolysates.

**Conclusion:**

Bacteriophage lytic enzymes are strong candidates for application as antimicrobials to control lactic acid bacterial contamination in fuel ethanol fermentations.

## Background

The fuel ethanol industry has experienced rapid growth in recent years, with 10.6 billion gallons of ethanol produced in 2009 and future need estimated to be 60 billion gallons by 2030 in the United States alone [1]. Currently, the majority of ethanol is produced from renewable carbohydrate-rich feedstock such as cornstarch or sugarcane, but to achieve higher demands in the future, lignocellulosic biomass will need to be utilized. Weakening the economics of biofuel production are ethanol losses due to bacterial contamination of fermentation cultures. Contributing to this concern is the fact that it is not feasible to produce fuel ethanol under aseptic conditions, therefore chronic and acute contaminations are commonplace [2-5]. A variety of Gram positive and Gram negative bacteria have been isolated from commercial fuel ethanol production facilities [3,5,6]. However, it is generally believed that lactic acid bacteria (LAB) are the most detrimental, with *Lactobacillus* species predominating [5-7]. Lactobacilli thrive in the industrial fermentation environments because they are well adapted for survival under the high ethanol, low pH and low oxygen conditions. A major culprit, *L. fermentum,* has been shown to reduce ethanol production in *Saccharomyces cerevisiae* fermentation cultures by as much as 27% [6,8]. Controlling LAB in fermentation cultures often requires prophylactic antibiotic treatments and/or costly production shutdowns for extensive cleaning and disinfecting [5,9,10]. Despite current control measures and practices, long-term suppression of microbial contamination is still a major challenge in ethanol production.

There are numerous theories to account for the effect contaminants have on yeast during ethanol production. Chronic LAB contaminants are believed to compete for sugars available for conversion to ethanol as well as essential micronutrients required for optimal yeast growth. Acute contaminations often lead to the accumulation of major inhibitory end-products such as acetic and lactic acid that inhibit yeast growth and, if left untreated, cause “stuck” fermentations [5,11,12]. Besides lowering the pH of the fermentation below the optimal *S. cerevisiae* pH range for the conversion of sugars to ethanol, the acid’s true inhibitory effect has been postulated to be from the undissociated form of the acid that is capable of diffusing through the yeast cell membrane where it dissociates, acidifying the cytoplasm [13]. Other compounds produced by LAB are known to contribute to the inhibition of ethanol production such as diacetyl [14], fatty acids [15] and the broad spectrum antibiotic reuterin [13,16].

Several techniques are currently being employed in an attempt to control microbial contaminants. In the United States, bacterial contaminants are commonly controlled with the commercially available antibiotics virginiamycin, penicillin, and erythromycin [5,9,10]. Treatment for contamination is often prophylactic, necessitating the addition of antibiotics to each fermentation cycle. However, decreased susceptibility to virginiamycin has already been observed in *Lactobacillus* species isolated from dry-grind ethanol plants that use virginiamycin [17] and the emergence of isolates with multidrug resistance to both virginiamycin and penicillin have also been reported [9,17]. In addition, concerns over the potential for antibiotic residues to persist in the distillers grains co-products may further limit their use during ethanol production [18]. A ‘no-antibiotic’ approach has obvious advantages, but acceptable alternatives are currently lacking.

**Figure 1:**
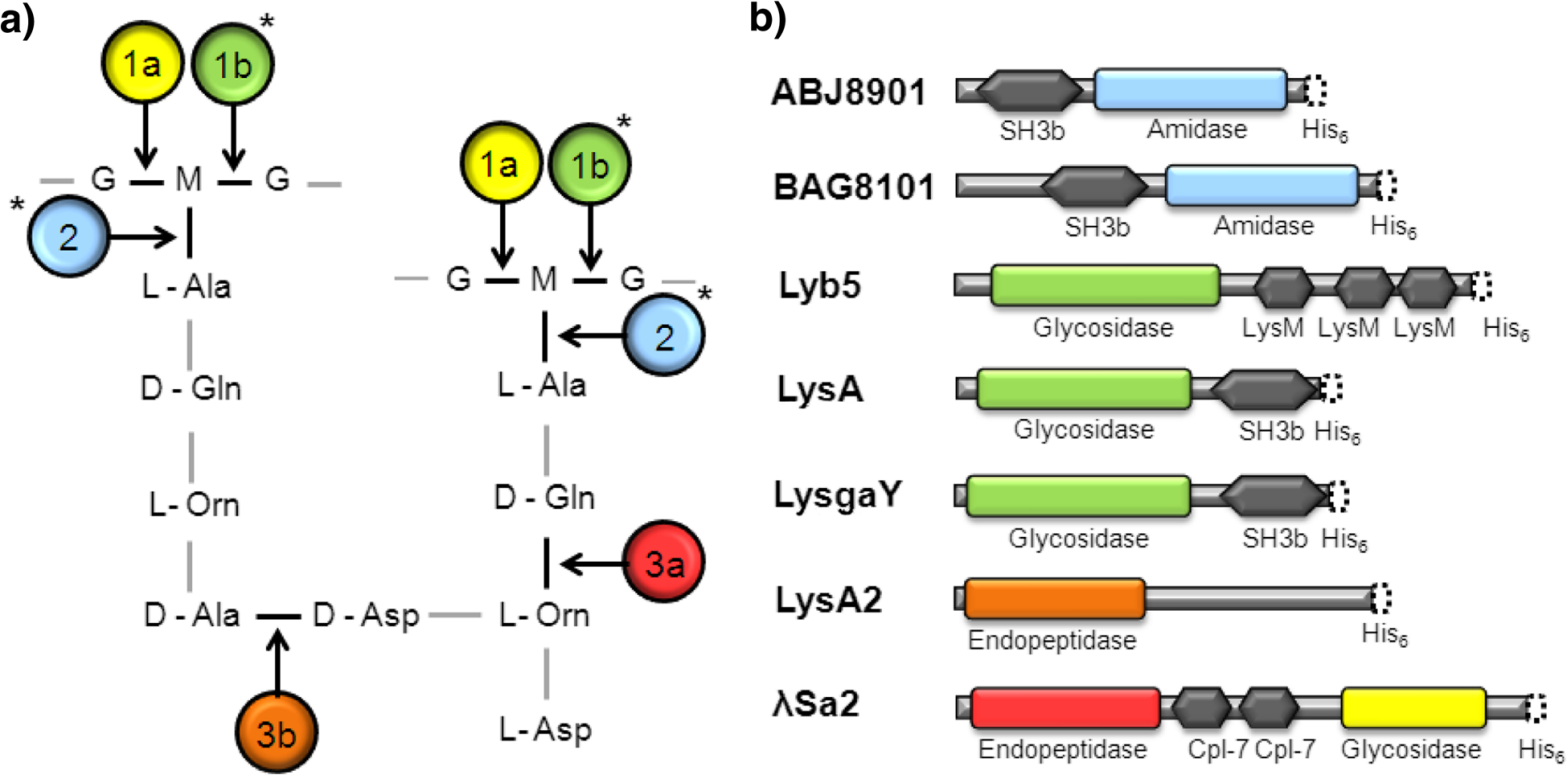
**Schematic representation of PG, putative lysin catalytic sites and domain structures of bacteriophage lysins. a)** Fragment of the repeat structure of *Lactobacillus fermentum* PG with a D-Asp interpeptide bridge with known enzymatic cut sites: (1a) λSa2 endolysin; (3a) λSa2 endolysin; (3b) LysA2, and predicted lysin catalytic sites: (1b*) Lyb5, LysgaY, and LysA; (2*) ABJ8901 [GenBank: ABJ63875] and BAG8101 [GenBank:BAG27815]. * Predicted catalytic sites are based on amino acid homologies to other biochemically characterized enzymes. **b)** Lysin architecture consisting of an enzymatically active domain(s) (square box) and cell wall binding domain(s) (hexagon box) drawn nearly to scale predicted using the NCBI Conserved Domain Database. A His_6_-tag (dot box; not to scale) was fused on the C-terminal for metal ion affinity chromatography purification.

Bacteriophage (phage) endolysins are lytic enzymes produced by bacterial viruses. During phage infection of bacteria, lysins are produced near the end of the phage replication cycle to degrade peptidoglycan (PG) (a major structural component of the bacterial cell wall) that leads to cell lysis (‘lysis from within’) and phage progeny release (reviewed in [19]). Scientists have found that externally lysin-treated bacteria still lyse (exolysis or ‘lysis from without’), which has been exploited to control pathogenic and problematic bacteria [20-23]; for review see [24]. Currently, lysins are only exolytic for Gram positive bacteria that lack an outer membrane, which prevents access of the lysin to the PG of Gram negative bacteria. Lysins exert their lethal effects by forming holes in the PG. This degradation of the cells wall, results in the extrusion of the cytoplasmic membrane due to the approximately 30 or 40 atm intracellular pressure resulting in osmolysis [24]. PG is unique to bacteria and has a complex structure [25] with a sugar backbone of alternating units of N-acetyl glucosamine and N-acetyl muramic acid. Typically, these sugar polymers are cross-linked by species-specific oligopeptide attachments at the N-acetyl muramic acid residues (Figure 1a). Phage lysins have evolved a modular design to deal with PG complexity, generally consisting of both lytic domains and cell wall binding (CWB) domains (Figure 1b). Catalytically, a single lysin molecule should be sufficient to cleave an adequate number of bonds to lyse a bacterial wall [26].

There are numerous candidate lactobacilli lysin genes available in both phage genomes and prophage genomes within public data sets. We have attempted to isolate and screen seven phage lysins for their ability to kill six *Lactobacillus* strains isolated from fuel ethanol fermentations and sixteen other Gram positive strains. Of these lysins, three lactobacilli lysins: LysA [27], LysA2 [28] and LysgaY [29], and a streptococcal phage LambdaSa2 (λSa2) lysin [30,31], were successfully expressed, purified and examined in this study. We demonstrate that LysA and λSa2 endolysin are highly exolytic against a variety of lactobacilli including the notorious *L. fermentum* contaminant, under laboratory conditions that mimic ethanol fermentation environments. These results suggest that lysins have the potential to control unwanted lactobacilli contaminations in fermentation systems.

## Results

### Lysin purification

**Figure 2:**
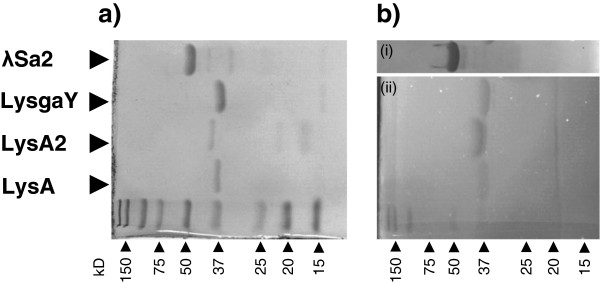
**SDS-PAGE and exolytic activity analyses of Immobilized Metal Affinity Chromatography purified recombinant lysins. a)** SDS-PAGE with high intensity bands correspond to predicted molecular weights for lysins λSa2 endolysin (51.9 kD), LysgaY (33.9 kD), LysA2 (37.4 kD), and LysA (36.4 kD). **b)** Zymogram analysis with whole cells substrate (i) *Lactobacillus reuteri* isolate 14171 and (ii) *Lactobacillus amylovorus* isolate 4540, co-polymerized within the polyacrylamide gel. Lysin exolytic activity resulted in visible clearing (dark bands) of the cell substrate at the point of protein localization, which corresponds with predicted molecular weights.

We were unable to purify three of seven cloned putative lytic proteins ABJ8901 [GenBank:ABJ8901], BAG8101 [GenBank: BAG8101] and Lyb5 from phiPBY5 [32], due to complications during IPTG induction of *E. coli* BL21(DE3) transformed cells. [After induction, the *E. coli* culture unexpectedly autolysed which led to culture failure (data not shown).] Induction and purification of putative lytic proteins λSa2 endolysin, LysgaY, LysA2, and LysA occurred without complication. SDS-PAGE analysis of the nickel chromatography purified proteins produced prominent bands for λSa2 endolysin, LysgaY, LysA2 and LysA that migrated to positions in the SDS PAGE consistent with their predicted molecular masses of 51.9 kD, 33.9 kD, 37.4 kD, and 36.4 kD, respectively (Figure 2a).

### Lysin exolytic activity and bacteria susceptibility spectrum

Zymogram analysis was performed with whole cells of *L. reuteri* 14171 or *L. amylovorus* 4540 co-polymerized within the polyacrylamide gel matrix (Figure 2b). Single translucent or dark bands, indicating lysis of the embedded cells, were observed in each lane at approx. 52 kD, 38 kD, 35 kD, and 37 kD, in agreement with the predicted MW of λSa2 endolysin, LysgaY, LysA2, and LysA, as indicated by SDS-PAGE (Figure 2a), respectively.

Purified lysins were tested in turbidity reduction assays for their ability to lyse log phase cultures of several bacterial species (Table 1). The λSa2 endolysin had strong lytic activity towards 17 of 22 lactobacilli, staphylococci or streptococci, and weaker activity towards another three. LysA, LysA2 and LysgaY had similar exolytic host ranges towards about 60% of the lactobacilli tested including all four *L. fermentum* isolates, and *L. gasseri*, *L. brevis* and *L. reuteri*. LysA was able to reduce cell populations >75% for 50% of the sensitive strains and >50% for the remaining strains tested. However, LysA2 and LysgaY were only able to decrease cellular turbidity by <50%. Interestingly, LysA and LysgaY had exolytic activity towards 100% of the streptococci and 80% of the staphylococci tested, whereas LysA2 did not show any exolytic activity towards the non LAB species tested. None of the lysins tested had exolytic activity towards the Gram negative *E. coli* DH5α or the yeast strain of *S. cerevisiae*.

**Table 1:** **Activity of LysA, LysA2, LysgaY and λSa2 endolysin against lactic acid bacteria as determined by turbidity (OD**_**600nm**_**) reduction analysis**

	**Activity**^**a**^				
**Strain**	**LysA**	**LysA2**	**LysgaY**	**λSa2**	**Strain Source**
**Gram positive**					
*Lactobacillus amylovorus* B-4540	-	++	++	++	NRRL^*b*^
*Lactobacillus brevis* 0605-48	++	++	++	++	*this study*^*c*^
*Lactobacillus delbrueckii* subsp *delbrueckii* B-763	-	-	+	+	NRRL
*Lactobacillus fermentum* BRO315-1	++	+	+	++	*this study*
*Lactobacillus fermentum* BRO315-25	++	+	+	+++	*this study*
*Lactobacillus fermentum* 0605-B44	+++	+	+	+++	*this study*
*Lactobacillus fermentum* 0713-3	+++	+	+	+++	*this study*
*Lactobacillus gasseri* B-4240	+++	+	+	+++	NRRL
*Lactobacillus hilgardii* B-1843	-	-	-	-	NRRL
*Lactobacillus malefermentans* B-1861	-	-	-	++	NRRL
*Lactobacillus paracasei* BRO315-44	-	-	-	-	*this study*
*Lactobacillus reuteri* B-14171	++	+	++	+	NRRL
*Staphylococcus aureus* Newman	+	-	+	+++	Jean C. Lee^*d*^
*Staphylococcus epidermidis*	+	-	++	+++	USDA
*Staphylococcus hyicus*	-	-	-	+	USDA
*Staphylococcus warneri*	+	-	+	+++	USDA
*Staphylococcus xylocus*	++	-	++	+++	USDA
*Streptococcus agalactiae*	++	-	++	+++	David Pritchard^e^
*Streptococcus dysgalactiae*	++	-	++	+++	USDA
*Streptococcus pyogenes*	+	-	+	+++	Dan Nelson^f^
*Streptococcus suis* 531-668	+++	-	+++	+++	Dan Nelson^f^
*Weisella viridescens* B-1951	-	-	+	+++	NRRL
**Yeast**					
*Saccharomyces cerevisiae* Y-2034	-	-	-	-	NRRL
**Gram negative**					
*Escherichia coli* DH5α	-	-	-	-	Invitrogen

*a* As measured by OD_600nm_, decrease of whole cell suspensions (initial OD_600nm_ = ~1) treated with lysin (10μM) for 30 min. “+++” between 100% and 75% decrease, “++” between 74% and 50% decrease, “+” less than 49% decrease, “−“no decrease, with respect to no lysin control.

*b* ARS Culture Collection, Peoria, IL (also known as the NRRL Collection).

*c* Isolated from fermentors at a commercial dry-grind ethanol facility as described previously [6]. *L. fermentum* BR0315-1, BR0315-25, and 0713–3 were planktonic isolates, and their MICs for virginiamycin are 16 μg/ml, ≤2 μg/ml, and ≤2 μg/ml, respectively. *L. fermentum* 0605-B44 was a biofilm isolate from coupon scrapings of a Centers for Disease Control biofilm reactor inoculated with a mash sample from the fermentor.

*d* Channing Lab, Womens and Brigham Hospital, Boston, MA.

*e* Dept. Biochemistry, Medical School, Univ. Alabama, Birmingham.

*f* Department of Veterinary Medicine, UMD, College Park MD.

### Sensitivity of exolytic activity to pH and ethanol

**Figure 3:**
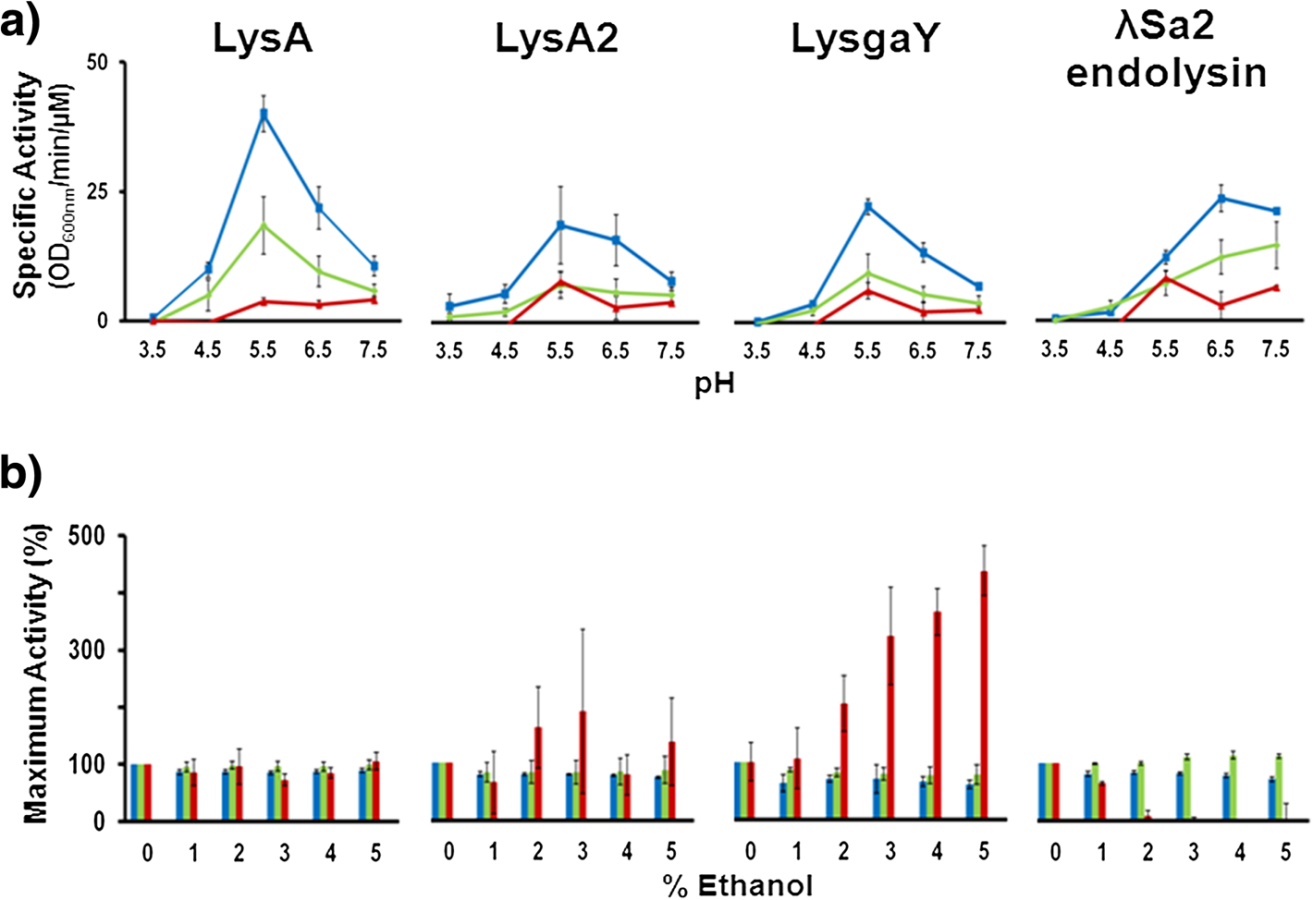
**Turbidity reduction analysis of LysA, LysA2, LysgaY and λSa2 endolysin against multiple lactobacilli in a range of pH and ethanol concentrations. a)** Effect of pH on turbidity reduction specific activities (OD_600nm_/min/μM; as described by [49]). **b)** Effect of ethanol on lysin activity (normalized to specific activity achieved at pH 5.5 in panel a). Live cells of *L. fermentum* isolate 0605-B44 (blue), *L. fermentum* isolate BR0315-1 (green), and *L. brevis* isolate 0605–48 (red) were used as substrate. Data represent the average of three experiments (n=3) ± SEM.

The functional properties for lysins LysA, LysA2, LysgaY and λSa2 endolysin were tested under a range of pH and ethanol concentrations with turbidity reduction analysis using live cells of *L. fermentum* isolates 0605-B44 and BR0315-1, and *L. brevis* isolate 0605–48, as substrate. Optimal specific activity was achieved for LysA, LysA2, and LysgaY at pH 5.5, although these lysins demonstrated strong exolytic activity between pH 5.5 and pH 6.5 (Figure 3a). For these three lysins, the presence of ethanol (≤5%) in the turbidity reduction assay did not reduce the maximum activity achieved (Figure 3b). However, LysgaY activity improved approximately 3-fold when *L. brevis* was used as substrate in the presence of 3% ethanol and nearly 5 fold at 5% ethanol. The streptococci phage λSa2 endolysin produced its optimal specific activity at a slightly higher pH of 6.5 and also was not affected by the presence of ethanol (≤5%) for both *L. fermentum* substrates. However, activity with *L. brevis* substrate in ≤1% ethanol completely abolished exolytic activity.

### Lysins are exolytic in the fermentation environment

**Figure 4:**
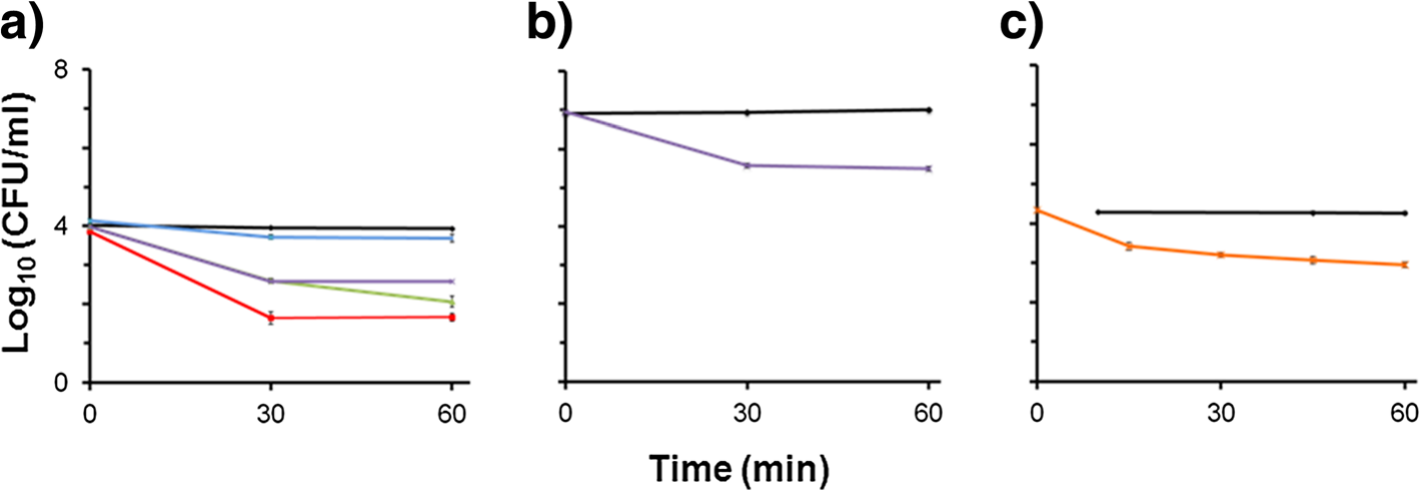
**λSa2 and LysA exolytic activity in mock fermentations of corn fiber hydrolysates inoculated with *L. fermentum *and *L. reuteri. *a)** Hydrolysate inoculated with 1x10^4^ CFU/ml *L. fermentum* isolate BR0315–1 and treated with λSa2 endolysin at 250 ng/μl (red), 75 ng/μl (green), 25 ng/μl (purple) and LysA at 25 ng/μl (blue), PBS buffer control (black). **b)** Hydrolysate inoculated with 1x10^7^ CFU/ml *L. fermentum* isolate BR0315-1 and treated with 25 ng/μl (purple) λSa2 endolysin, PBS buffer control (black). **c)** Hydrolysate inoculated with 1x10^4^ CFU/ml *L. reuteri strain* B-14171 and treated with LysA at 760 ng/μl (orange), PBS buffer control (black). Data represent the average of four plate counts (n=4) ± SEM.

Industrial fermentation substrates have high sugar concentrations, and, for hydrolysates of lignocellulosic biomass, may also contain lignin degradation products (e.g. phenolic acids, acetic acid, and furfural). To test whether this environment may inhibit our lysins, we tested the efficacy of the lysin λSa2 and LysA to reduce viable *L. fermentum* (BR0315-1) and *L. reuteri* (B-14171) in mock fermentations of corn fiber hydrolysates. λSa2 endolysin was tested at a final concentration of 250ng/μl, 75ng/μl, and 25ng/μl in a fermentation experimentally inoculated with 1x10^4^ CFU/ml *L. fermentum.* Bacterial loads in cultures treated with λSa2 endolysin decreased within 30 min, and by 60 min, reductions in bacterial concentration ranged from ~1.5 log_10_ (CFU/ml) at 25 ng/μl λSa2 endolysin to ~2.5 log_10_ (CFU/ml) at 250ng/μl λSa2 endolysin (Figure 4a). LysA had diminished exolytic activity compared to λSa2 endolysin, generating a reduction of only ~0.45 log_10_ (CFU/ml) in hydrolysates containing 1x10^4^ CFU/ml (Figure 4a). LysA was also tested against *L. reuteri* to demonstrate lysin abilities to kill other *Lactobacillus* species (Figure 4c). In hydrolysates spiked with 4 log_10_ (CFU/ml) *L. reuteri*, LysA was able to reduce inoculum by ~1.39 log_10_ (CFU/ml) (Figure 4c).

Since contaminating bacterial loads in commercial fermentation cultures may reach 10^6^ to 10^8^ CFU/ml [3], the λSa2 endolysin (25 ng/μl) was tested against 10^7^ CFU/ml *L. fermentum* BR0315-1. After 60 min, λSa2 endolysin reduced bacterial load from 7.0 log_10_ (CFU/ml) to 5.5 log_10_ (CFU/ml) (Figure 4b)**,** a similar level of reduction shown in the mock fermentation contaminated with 1x10^4^ CFU/ml *L. fermentum* (Figure 4a). Untreated mock fermentations did not yield any reduction in contamination.

## Discussion

The goal of this study was to identify phage exolytic lysins that can be used as antimicrobials toward Gram positive LAB known to contaminate fuel ethanol fermentations. From seven putative lysin genes, we have identified four phage lysins (LysA, Lysa2, LysgaY and λSa2 endolysin), that show high activity against LAB contaminants from fuel ethanol facilities. These enzymes have broad exolytic activity *in vitro* towards numerous Gram positive LAB including several fermentation isolates of *L. fermentum*. Although LysA, LysA2 and LysgaY, and λSa2 endolysin all demonstrated exolytic activity against lactobacilli, the lactobacilli lysin LysA and the streptococcal λSa2 phage endolysin showed the greatest efficacies to reduce populations of *L. fermentum*. Interestingly, λSa2 endolysin also exhibited the broadest lytic activity towards the LAB and other Gram positive bacteria tested here.

It is virtually impossible to avoid LAB contaminations in fuel ethanol fermentations, and therefore, the risk of reduced ethanol yield is a major concern. The most common commercially available products used to control contamination in fuel ethanol facilities are based on the antibiotics virginiamycin and penicillin [2,9] with recommended dosages ranging in fuel ethanol fermentations between 0.25–2.0 ppm [10] which makes the fuel ethanol industry one of the largest consumers of antibiotics. However, the emergences of antibiotic resistant lactobacilli have occurred in fuel ethanol production facilities [9,17]. Phage lysins can avoid many resistance pitfalls associated with antibiotic use. Typically antibiotic resistance is a consequence of a bacterial mutation or acquisition of genes that improve the fitness of the recipient bacterium allowing it to evade the action of antibiotics. These adaptations generally occur inside the bacterial cell and employ three general strategies; modification of the drug, alteration of the target (or its level of expression), or decreased accessibility of the drug to its target (reviewed by Bischoff et al. [33]). Whereas, phage lysins target the PG, which is located outside the cytoplasmic membrane and reduce the number of possible known mechanisms by which bacterial resistance typically emerges [34].

Lysins are currently being used as disinfectants in industrial settings. Lysozyme which is isolated from hen egg albumen is also a PG hydrolytic enzyme similar to phage encoded lysins. It has been found to be useful in controlling unwanted bacteria in wines at concentrations of 250–500 mg l^−1^[35]. Although lysozyme has been found to inhibit undesirable malolactic fermentation by *Oenococcos oeni*, strains of pediococci and lactobacilli, which are usually blamed for serious defects in musts and wines, were resistant [36]. Bacteria are also known to produce various PG hydrolases, for example, a *Streptomyces* strain produces a mixture of muramidases and proteases that are secreted into the medium. When collected, this mixture has shown broad lytic activity against a variety of wine-relevant LAB, however lytic activity on *L. fermentum* was not tested [37] therefore it is uncertain if this approach could be used in a fuel ethanol fermentation system. Other examples for the potential use of lysin-based environmental disinfectants include lysins PlyC and Lysostaphin [38]. The streptococcal lysin PlyC was found to be 1,000 fold more active on a per weight basis than a commercially available oxidizing disinfectant and was shown to retain effectiveness when tested in the presence of non-ionic detergents, hard water, and organic material [38]. The staphylococcal lysin lysostaphin, a PG hydrolase bacteriocin, has been shown to be effective in killing methicillin resistant *S. aureus* (MRSA) on solid surfaces [39]. Therefore the study of phage-encoded lytic enzymes with activity against problematic LAB in fuel ethanol fermentations is highly relevant and needed.

The differences we observed in exolytic activity of the four lysins against different species Table 1, are possibly reflected in compositional changes in the cell walls. Of the three lactobacilli lysins tested, the exact cut site has only been experimentally determined for LysA2. LysA2 cleaves the bond established between the bridging aspartic acid and the final D-alanine of one of the tetrapeptides involved in the binding of adjacent PG chains of *L. casei* (Figure 1b) [28]. This particular architecture is typical of lactobacilli as well as many other LAB, including the pediococci [25]. However, some lactobacilli were only moderately exolysed by LysA2 (*L. fermentum, L. reuteri,* and *L. gasseri*) while others (*L. delbrueckii, L. malefermentans, L. paracasei*) were not sensitive to the lysin. A plausible explanation for this lysin specificity could be derived from the cell wall binding domain. Certain *Listeria monocytogenes* lysins have cell wall binding domains that specifically recognize and bind to teichoic acids before degradation of the peptidoglycan can occur [40]. For the lysins tested here, it is not known if and how the cell wall binding domains are interacting with the cell wall or the prevalence of potential cell wall binding epitopes on the cell surface of the LAB tested in this study. Similarly, ethanol stresses on the cell wall (Figure 3b) or phase of growth (logarithmic, stationary, or biofilm) may contribute significantly to sensitivity to lysins, although lysins are generally believed to be active on all phases of cell growth.

Interestingly, the streptococcal enzyme, λSa2 prophage endolysin, was our strongest candidate antimicrobial for lactobacilli. The most likely explanation for this cross-genera activity is that the λSa2 endolysin glycosidase lytic domain targets the conserved sugar backbone common to all PG. This C-terminal N-acetyl-glucosaminidase cleaves the glycan component of the PG on the reducing side of GlcNAc (Figure 1a) [30]. The N-terminal λSa2 endolysin catalytic domain harbors a D-glutaminyl-L-lysine endopeptidase, which cleaves the peptide bonds between the two amino acids D-glutamine and L-lysine [30]. This exact sequence is present in most lactobacilli PG, but is lacking in the PG of *L. fermentum* (Figure 1a), the species where λSa2 endolysin is apparently most active (Table 1). *L. fermentum* has an L-ornithine substituted for L-lysine at the third position of the tetrapeptide (Figure 1a). Although we have no biochemical data to indicate which domain (glycosidase or endopeptidase) is responsible for the lysis of the LAB strains, it is possible that the λSa2 endolysin endopeptidase domain functions to cleave this bond in LABs, when considering the similarity between ornithine and lysine (lysine harbors 4 carbons, rather than 3 for ornithine in the amino terminal alkyl side chains). In support of this possibility is the fact that LysA2 endopeptidase activity [hydrolyzes the bond between the terminal D-alanine of the PG tetrapeptide and the D-aspartic acid residue that forms the bridge with the L-lysine of a neighboring PG chain] was reported to function on species that harbor either an L-lysine or an L-ornithine at position three in the neighboring tetrapeptide [28]. Although not a definitive or even a direct comparison, this suggests a degree of flexibility in the recognition sequences surrounding the cut site of these PG hydrolase lytic domains.

It is interesting to speculate on whether or not sensitivity to ethanol will be a significant factor in the efficacy of the lytic enzymes we have tested. Although, final fermentation conditions yield ethanol concentrations that may be greater than 5%, the starting feedstock is a major culprit for introducing LAB contamination to the fermentation system [4], a point at which the fermentation is highly sensitive to contamination [41]. At this initial aerobic stage in the fermentation there is no significant ethanol concentration that would be ideal for lysin treatment therefore ethanol levels might not be a factor. Certainly enzymes that have a broad range of activity regardless of ethanol concentration might provide a longer-lived protection during the fermentation process, especially if they are designed to be secreted from recombinant fermentative yeast throughout the fermentation process. Yet to be determined is whether a broader species-target range or biochemical resilience under ethanolic fermentation conditions is more important for the optimal antimicrobial when considering the complex environment of a lignocellulosic fermentation. The increased activity of LysA2 and LysgaY against *L. brevis* with increasing ethanol concentration is intriguing and suggests these might be preferred enzymes if treating *L. brevis* contaminants in late fermentations.

From this study, LysA, LysA2, LysgaY and λSa2 endolysin were shown to be excellent candidate antibacterial agents. By homology screening of these lysins to other known PG hydrolase lytic and cell wall binding domains there is no shortage of phage lysins for future consideration as public datasets contain numerous putative lytic PG hydrolases from both bacterial (prophage) and phage genome origins. Due to the high interest in phages that impinge on yogurt production, there are hundreds of known lactobacilli phage, with nine complete *Lactobacillus* phage genomes and 11 *Lactobacillus* poly-lysogenic bacterial genomes with sequence available on the NCBI website [42]. For example, lactobacilli lysins from Φadh [43], and Φg1e [44,45] have been shown to be functional lytic enzymes, although their ability to exolyse cells has not been reported. There is also a report of an amidase domain from the PL-1 that infects *L. casei*[46] and a muramidase (Mur-LH) that have shown a broad species activity ([47]. However, due to limitations in the species range of lytic activity each candidate will need to be tested empirically against target LAB.

Our most broadly effective enzymes, λSa2 endolysin and LysA, were tested in mock fermentations and shown to effectively reduce the LAB load by up to 2 orders of magnitude. However promising these results may seem, these initial trials do not necessarily reflect normal fermentation conditions. In order to easily detect changes in target contaminant profiles, the mock trial was performed under pre-sterilization conditions, such that the only contaminant was *L. fermentum*, a scenario not likely in industrial fermentations. Also, neither the yeast nor the LAB were adapted for growth on hydrolysate, rather, they were both grown in rich broth and then added to the hydrolysate. This assay was designed to test the enzymes for activity under hydrolysate conditions, the results of which are encouraging, but activity on the contaminant might be affected by the cell wall of the contaminant during hydrolysate growth conditions. Thus, the usefulness of these enzymes in large scale fermentations remains to be determined.

Due to the absence of PG in yeast cell walls, none of these lysins including λSa2 endolysin had any catalytic activity towards *S. cerevisiae* when applied externally and should not adversely affect the fermentation process. In addition, the heat of distillation and the heat of drying the distiller’s grains should denature the lysins, minimizing any potential impact on gut microflora of animals fed the ethanol co-products. Therefore, lysins appear to share qualities worth considering for future works to protect fuel ethanol fermentations from LAB contaminants.

## Conclusions

Phage lytic enzymes are strong candidate antimicrobials to control LAB contamination in fuel ethanol fermentations. Four phage endolysins of Gram positive origin (LysA, LysA2, LysgaY and λSa2 endolysin) demonstrate lysis of LAB at pH and ethanol concentrations similarly achieved during fuel ethanol fermentations. Two of these enzymes (λSa2 endolysin and LysA) reduce LAB by at least one log in mock fermentations. These qualities make phage lytic enzymes excellent candidate antimicrobials for testing in biofuel fermentations as either additives or engineered to be expressed by the fermentative yeast.

## Methods

### Constructs, strains, and plasmids

The lysins that were bioinformatically selected and synthesized or generously gifted to us for use in this study are listed in Table 2. LysA, LysA2, LysgaY genes were bioinformatically reverse translated with an *E. coli* codon bias and gene nucleotide sequences were commercially synthesized and cloned into pUC57 vector (GenScript, Piscataway, NJ). The *Streptococcus agalactiae* phage λSa2 endolysin (EMD Biosciences, San Diego, CA) was obtained as a gift from D. Pritchard [30]. LysA, LysA2, LysgaY and λSa2 endolysin genes were sub-cloned into the pET21a *E. coli* expression vector (EMD Biosciences, San Diego, CA) and maintained in *E. coli* DH5α (Invitrogen, Carlsbad, CA) at 37°C in LB medium supplemented with 150 μg/ml ampicillin for plasmid purification, maintenance and DNA sequence verification.

**Table 2:** Lysins bioinformatically selected from GenBank for cloning into pET21a protein expression vector

**Lysin**	**Origin**	**Reference or Source**
ABJ8901	*Lactobacillus brevis* ATCC367	[GenBank:ABJ63875]
BAG8101	*Lactobacillus fermentum* IFO 3956	[GenBank:BAG27815]
Lyb5	*Lactobacillus fermentum phage* ΦPBY5	[32]
LysA	*Lactobacillus fermentum* Br11	[27]
LysA2	*Lactobacillus casei* phage ΦA2	[28]
LysgaY	*Lactobacillus fermentum phage* ΦgaY	[29]
λSa2 endolysin	*Streptococcus agalactiae*	[30]

The bacterial strains used and their origin are listed in Table 1. Staphylococci and streptococci were grown in Tryptic Soy Broth (TSB; Difco Laboratories) medium with shaking at 37°C, *E. coli* was grown in Luria-Bertani (LB; Difco Laboratories) medium broth at 37°C with shaking, and lactobacilli were grown in de Man, Rogosa and Sharp (MRS; Difco Laboratories) medium at 37°C without shaking. *S. cerevisiae* was grown overnight in yeast extract peptone (YP; Becton, Dickinson, and Co. Sparks, MD) medium supplemented with 4% (w/v) glucose at 30°C with shaking.

### Expression and purification of lysins

All lysin proteins were over expressed in *E. coli* and purified via nickel column chromatography as previously described [48]. Purified pET21a harboring the lysin genes of interest, were transformed into *E. coli* BL21 (DE3) (Invitrogen, Carlsbad, CA) by heat stock at 42°C for 30 s. BL21 (DE3) transformants were cultured at 37°C in 1 L modified LB (mLB) medium (15 g/l tryptone, 8 g/l yeast extract, 5 g/l NaCl) supplemented with 150 μg/ml ampicillin. Mid log phase (OD_600 nm_ of 0.4–0.6) cultures were induced with 1 mM IPTG (isopropyl-beta-D-thiogalactopyranoside), followed by 10°C overnight incubation. Induced cells were pelleted, resuspended in 10 ml of lysis buffer (50 mM NaH_2_PO_4_, 300 mM NaCl, 10 mM imidazole, 30% glycerol, pH 8.0), and sonicated on ice for 15 min (1 s pulses separated by 1 s rests). After centrifugation (9000 x g for 30 min), proteins were purified from the cleared supernatant by immobilized metal ion affinity chromatography, using nickel-NTA Superflow resin (QIAGEN, Valencia, CA). Resin was washed with 40 column volumes (CV) of lysis buffer, and 15 CV of wash buffer (50 mM NaH_2_PO_4_, 300 mM NaCl, 20 mM imidazole, 30% glycerol, pH 8.0). His_6_-tagged proteins were eluted with elution buffer (50 mM NaH_2_PO_4_, 300 mM NaCl, 250 mM imidazole, 30% glycerol, pH 8.0) to achieve >90% purity. Protein elutes were filter sterilized (0.22 μm), concentration measured spectrophotometrically using a NanoDrop ND-1000 (NanoDrop Technologies, Wilmington, DE), and purities were determined via sodium dodecyl sulfate-polyacrylamide gel electrophoresis (SDS-PAGE).

### Zymogram and turbidity reduction assays

The purified proteins and Kaleidoscope protein standards (Bio-Rad) were analyzed using 15% SDS-PAGE, with or without 300 ml equivalent of mid log phase (OD_600 nm_ = 0.4–0.6) lactobacilli cells that were pelleted, and washed twice in buffer (50 mM NaH_2_PO_4_, 150 mM NaCl, pH 8.0) prior to addition to the pre-polymerized gel. Gels were electrophoresed at 150 volts (~1 h). SDS-PAGE gels were Coomassie stained and zymograms were washed in excess H_2_O for 1 h and incubated at 24°C in 50 mM Tris–HCl, 1% Triton X114, pH 5.5, until visible translucent bands appeared and images taken (~18 h).

The turbidity assays were performed in a Molecular Devices Spectra Max 340 plate reader. Strains were grown to mid-log phase (OD_600 nm_ = 0.4 – 0.6) at 37°C, washed in buffer (20mM phosphate, 150mM NaCl, 30% glycerol: pH 8.0), pelleted, and frozen at −80°C. Cells were thawed on ice and resuspended to OD_600 nm_ = 2.0 in buffer (20mM phosphate, 150mM NaCl: pH 5.5, unless otherwise stated). Lysins were standardized to 1 μM per well and the assay started by the addition of 100 μl of cell suspension, giving an initial OD_600nm_=1. Immediately, absorbance (OD_600nm_) readings were taken every 30 s for 30 min and specific activities were determined on a sliding scale as described by Becker et al. [49] as OD_600nm_/min/μM. Control samples with cells alone (no enzyme) were included, and ‘cells alone’ specific activities were subtracted from experimental sample specific activities control for the effect of autolysis.

### Preparation of mock fermentation

Corn fiber, obtained from a commercial wet-mill ethanol facility, was hydrolyzed by dilute acid treatment as previously described [50]. Briefly, sulfuric acid (1% w/v) was added to a suspension of corn fiber (10% w/v) and heated to 121°C for 1 h, then neutralized to pH 5.5 with NaOH. The hydrolysate was cleared of particulate matter by centrifugation. Sterility was confirmed by plating aliquots of hydrolysate on MRS agar. The *S. cerevisiae* strain NRRL Y-2034 was grown overnight in YP broth supplemented with 4% (w/v) glucose at 32°C with shaking at 200 rpm. Cells were harvested by centrifugation and resuspended in a volume of phosphate buffered saline (PBS; Fisher Scientific) pH7.4, necessary to obtain an OD_600nm_ equivalent of 80. *L. fermentum* strain BR0315-1 and *L. reuteri* strain B-14171 were grown to mid log phase (OD_600nm_ = 0.4-0.6) (as described above), diluted appropriately in PBS to a density of 1x10^5^ CFU/ml or 1x10^8^ CFU/ml (OD_600nm_ =1.0 is ~4.5x10^8^ CFU/ml) and used as inoculums for mock fermentation analysis.

Mock fermentation cultures were prepared by combining 10 ml of corn fiber hydrolysate, 100 μl 12% (NH_4_)_2_SO_4_, 150 μl cellulase (Celluclast; Novozymes, Bagsvaerd, Denmark), 150 μl Cellobiase (Novozyme 188; Novozymes), and 125 μl *S. cerevisiae* (OD_600nm_ equivalent of 80), in a 50 ml conical tube. For each lysin treatment analysis, 800 μl of mock fermentation prep was spiked with 100 μl mid log phase LAB cells (1x10^5^ CFU/ml or 1x10^8^ CFU/ml), and treated with 100 μl purified His_6_-tagged lysin. ‘No-lysin’ controls were included. Mock fermentations were cultured at 30°C with aliquots taken at 0 min, 30 min, and 60 min intervals. The aliquots were titered by plating on 1.5% MRS agar containing 100 μg/ml cyclohexamide (to inhibit growth of *S. cerevisiae*) and incubated anaerobically using the AnaeroPack System (Mitsubishi, Tokyo, Japan) at 37°C for 24 h.

## Abbreviations

TSB: Tryptic Soy Broth; LB: Luria-Bertani; mLB: modified Luria-Bertani; MRS: de Man, Rogosa and Sharp; YP: Yeast extract peptone; PBS: Phosphate buffered saline; IPTG: Isopropyl-β-D-thiogalactoside; LAB: Lactic Acid Bacteria; CFU: Colony Forming Unit; PG: Peptidoglycan; OD: Optical Density; SDS-PAGE: Sodium Dodecyl Sulfate-Polyacrylamide Gel Electrophoresis; SEM: Standard Error of Means.

## Competing interests

The authors declare there are no competing interests.

## Authors’ contributions

DRR designed and performed experiments involving phage lysins, purification and characterization of exolytic activities by zymogram and turbidity reduction assays, and was the lead author of this manuscript. PAK performed mock fermentation experiments and assisted in writing and editing of this manuscript. KMB, SRH and DMD contributed to the design, coordination and assisted in all experimental work, and in the writing and editing of the manuscript. All authors read and approved the final manuscript.

Mention of a trade name, proprietary product or vendor does not constitute a guarantee or warranty of the product by USDA or imply its approval to the exclusion of other suitable products or vendors.
